# Relations of lipid parameters, other variables with carotid intima-media thickness and plaque in the general Chinese adults: an observational study

**DOI:** 10.1186/s12944-018-0758-9

**Published:** 2018-05-10

**Authors:** Qingtao Hou, Sheyu Li, Yun Gao, Haoming Tian

**Affiliations:** 1 Department of Geriatrics, The First Affiliated Hospital of Chongqing Medical University, Chongqing, 400016 China; 2 West China School of Medicine, Sichuan University, Chengdu, 610041 China; 3 Department of Endocrinology and Metabolism, West China Hospital, Sichuan University, Chengdu, 610041 China

**Keywords:** Carotid intima-media thickness, Carotid plaque, Lipids, Chinese adults

## Abstract

**Background:**

It has been reported that non-high-density lipoprotein cholesterol (non-HDL-C) and lipid ratios, including total cholesterol (TC) / high-density lipoprotein cholesterol (HDL-C) and low-density lipoprotein cholesterol (LDL-C) / HDL-C, are better predictors for atherosclerosis than conventional lipid profiles. However, there have been few studies comparing the predictive values of different lipid parameters for early atherosclerosis. The aim of this study was to determine the relevant factors of carotid intima-media thickness (IMT) and plaque in the general Chinese adults and analyze the predictive values of different lipid parameters for carotid IMT and plaque.

**Methods:**

We collected the demographics, anthropometrics, and laboratory data of 311 Chinese adults without the diagnoses of acute myocardial infarction, stroke, heart failure, peripheral arterial disease, end-stage renal disease or malignant tumor. The carotid IMT and the presence of carotid plaque were evaluated by high-resolution color Doppler ultrasonography.

**Results:**

Based on the cutoff level of 0.9 mm, the percentage of people with a thickened IMT was 8.4%. And the percentage of people with carotid plaque was 15.8%. Among the lipid parameters, the levels of TC, non-HDL-C and LDL-C were more closely related to carotid IMT and plaque compared with other lipid parameters in the univariate analyses. In multivariate analyses, age, gender and systolic blood pressure (SBP) remained significantly with carotid IMT, whereas age, gender, diastolic blood pressure (DBP) and the TC level remained significantly with carotid plaque. Non-HDL-C level remained significantly with carotid plaque after adjusting for age, gender, waist-hip ratio (WHR), smoking, drinking, SBP and fasting plasma glucose (FPG).

**Conclusions:**

Age, gender, SBP are important predictors for carotid IMT. Age, gender, DBP and TC are important predictors for carotid plaque. TC, LDL-C and non-HDL-C have greater predictive values for IMT and the presence of carotid plaque compared with other lipid parameters, among which TC has the greatest predictive value for the presence of carotid plaque. The predictive value of non-HDL-C for carotid IMT and plaque is not inferior to that of LDL-C.

## Background

Atherosclerosis plays a key role in the development of ischemic cardiovascular disease (CVD) and stroke. Dyslipidemia is a fundamental risk factor of atherosclerosis. Previous studies have reported that low-density lipoprotein cholesterol (LDL-C) is a classical atherogenic lipoprotein, while high-density lipoprotein cholesterol (HDL-C) is anti-atherogenic [1]. Recently, a number of prospective epidemiological studies have indicated that triglyceride (TG) level is positively correlated with the morbidity and mortality of coronary heart disease (CHD) [2, 3]. Although LDL-C has been considered as the primary target of lipid-lowering therapy, non-high-density lipoprotein cholesterol (non-HDL-C), calculated as total cholesterol (TC) minus HDL-C, has been recommended as the secondary target of lipid-lowering treatment, especially for those with high TG levels [1]. Unlike LDL-C, non-HDL-C is independent of lipoprotein composition [4]. Thus, non-HDL-C has shown a better predictive value for atherosclerosis and CVD than LDL-C in some researches [5]. In addition, lipid ratios, including TC/HDL-C and LDL-C/HDL-C ratios have been suggested as better predictors for atherosclerosis than each independent lipid parameter in some studies [6, 7]. To our knowledge, there have been limited data to validate the predictive values of different lipid parameters for early-stage asymptomatic atherosclerosis before developing into ischemic CVD and stroke.

The carotid artery is regarded as the window of systemic atherosclerosis due to its superficial location. Carotid intima-media thickness (IMT) and plaque are crucial predictors for ischemic CVD and stroke, particularly in the subclinical stage [8]. Many heart attacks and strokes occur suddenly in people without any clinical symptoms. Therefore, preventing the diseases before they develop at the early stage of atherosclerosis in people at the average risk-factor level will be an effective approach. Many risk factors for CVD and stroke have been identified, but data on their predictive values for early-stage asymptomatic atherosclerosis remain limited.

The purpose of the present study was to detect the relevant factors of carotid IMT and the presence of carotid plaque in the general Chinese adults and analyze the predictive values of different risk factors, including different lipid parameters, for carotid IMT and the presence of carotid plaque.

## Methods

### Participants

The participants came from a prospective epidemiological survey from 2007 to 2009 which aimed to investigate the prevalence of diabetes and cardiovascular diseases in Chinese. The study design was previously described elsewhere [9]. All participants were from a single community in Chengdu, Sichuan Province in 2009 and underwent a routine clinical examination, including physical examination, biochemical examination and carotid artery color Doppler ultrasound examination. Participants with a diagnosis of acute myocardial infarction, stroke, heart failure, peripheral arterial disease, end-stage renal disease or malignant tumor were excluded. We also excluded participants who were on lipid-lowering therapy.

### Demographic and clinical characteristics

Demographic data such as age, gender, nationality and history of alcohol and smoking were obtained from a previously well designed questionnaire. Smoking was defined as having smoked at least 100 cigarettes during their lifetimes. Drinking was defined as having consumed at least 30 g of alcohol per week for more than one year. Height, weight, waist circumference and hip circumference were manually measured. Body mass index (BMI) was calculated by the eq. BMI = weight (kg) / height (m) ^2^. Waist hip ratio (WHR) was calculated by the eq. WHR = waist circumference (cm) / hip circumference (cm). Blood pressure was measured with a standard sphygmomanometer in a sitting position after a ten-minute rest in the fasting state and presented as a mean value of three readings (in mmHg). Hypertension was defined as a systolic blood pressure (SBP) higher than 140 mmHg and/or diastolic blood pressure (DBP) higher than 90 mmHg or taking antihypertensive drugs. Diabetes mellitus (DM) was defined according to the revised 1999 WHO criteria: fasting plasma glucose level (FPG) ≥126 mg/dl (7.0 mmol/L); plasma glucose level ≥ 200 mg/dl (11.1 mmol/L) 2 h after a 75 g oral glucose load. Those who were taking antidiabetic medication were also defined as diabetic patients.

### Laboratory examination

Venous blood samples were collected from each participant in the early morning after a 12 h fast. FPG, TC, TG, HDL-C, alanine aminotransferase (ALT), aspartate aminotransferase (AST), blood urea nitrogen (BUN), creatinine (Cr) were measured in the central laboratory. LDL-C was directly measured by standard enzymatic methods (Roche Diagnostics) when TG ≥4.52 mmol/L. The Friedwald eq. (LDL-C = TC-HDL-C-TG/2.2) was used when TG < 4.52 mmol/L [10].

### Carotid artery color Doppler ultrasound

A high-resolution vascular ultrasound (Philips ATL 3500 device) was used to measure both the left and right common carotid arteries by the sonographers who were blinded to the participants’ clinical characteristics. IMT was defined as the distance between the leading edge of the lumen-intima and the leading edge of the media dventitia echo [11]. The larger IMT of bilateral common carotid arteries was taken as the final value of IMT. IMT > 0.9 mm was defined as thickened IMT [12].

### Statistical analysis

All the statistical analyses were performed by using SPSS 19.0 (IBMSPSS, Chicago, USA). Continuous variables were presented as mean ± standard deviation (SD) or percentiles, while categorical variables were presented as percentages. The Kolmogorov-Smirnov test was used to determine the normality of distributions and the abnormal distributed data were log-transformed. Student’s t-test, Mann-Whitney U test or Chi-square test was used to compare the data between groups as appropriate. Linear correlations between IMT and clinical characteristics (including lipid parameters) were evaluated by Spearman rank correlation. Univariate linear regression models and stepwise multiple linear regression models were conducted to examine relevant factors of carotid IMT. Univariate logistic regression models and multivariate logistic regression models (Backward LR) were performed to explore the independent predictors for carotid plaque. Additionally, the independent association of non-HDL-C level with carotid plaque was analyzed by four multivariate logistic regression models after adjusting for clinical variables. The results were considered significant when *p* < 0.05.

## Results

### Baseline characteristics

A total of 311 Chinese adults consisted of 111 men and 200 women, aged 23–78 years (mean age 48.25 ± 12.86 years) were enrolled. Baseline characteristics are presented in Table 1. The median IMT was 0.70 mm and carotid plaque was detected in 49 participants (15.8%). A total of 21 participants (6.8%) were diabetic patients, sixteen of which (76.2%) received insulin or oral antidiabetic drugs. Forty-eight participants (15.4%) were hypertensive patients, thirty-five of which (72.9%) received antihypertensive drugs. Of the participants without lipid-lowering therapy, thirty-one (10.0%) had dyslipidemia.

**Table 1:** Baseline characteristics of participants

Characteristics	Mean ± SD or P50 (P25, P75) or number (percentage)
Age (years)	48.25 ± 12.86
Male, n (%)	111 (35.7)
Han, n (%)	309 (99.4)
BMI (kg/m^2^)	23.24 (20.90, 25.80)
WHR	0.88 ± 0.08
Smoking, n (%)	67 (21.5)
Drinking, n (%)	81 (26.0)
Diabetes, n (%)	21 (6.8)
Hypertension, n (%)	48 (15.4)
Dyslipidemia, n (%)	31 (10.0)
Antidiabetic treatment, n (%)	16 (5.1)
Antihypertensive treatment, n (%)	35 (11.3)
FPG (mmol/L)	4.82 (4.54, 5.18)
SBP (mmHg)	114.00 (102.67, 129.33)
DBP (mmHg)	75.97 ± 10.98
TC (mmol/L)	4.78 ± 0.91
TG (mmol/L)	1.03 (0.80, 1.52)
LDL-C (mmol/L)	2.78 (2.27, 3.31)
HDL-C (mmol/L)	1.60 ± 0.34
Non-HDL-C (mmol/L)	3.18 ± 0.90
TC/HDL-C	2.94 (2.54, 3.46)
LDL-C/HDL-C	1.79 (1.41, 2.23)
ALT (IU/L)	19.00 (15.00, 28.00)
AST (IU/L)	22.00 (19.00, 27.00)
BUN (mmol/L)	5.39 (4.34, 6.54)
Cr (umol/L)	55.44 ± 14.56
IMT (mm)	0.70 (0.60, 0.80)
Carotid plaques, n (%)	49 (15.8)

*SD* Standard deviation, *BMI* Body mass index, *WHR* Waist-hip ratio, *FPG* Fasting plasma glucose, *SBP* Systolic blood pressure, *DBP* Diastolic blood pressure, *TC* Total cholesterol, *TG* Triglyceride, *LDL-C* Low-density lipoprotein cholesterol, *HDL-C* High-density lipoprotein cholesterol, *Non-HDL-C* Non-high-density lipoprotein cholesterol, ALT Alanine aminotransferase, *AST* Aspartate aminotransferase, *BUN* Blood urea nitrogen, *Cr* Creatinine, *IMT* Intima-media thickness

### Characteristics classified based on the presence of carotid plaque

As demonstrated in Table 2, the levels of TC, LDL-C, HDL-C and non-HDL-C in the subjects with carotid plaque were significantly higher as compared with those in the subjects without carotid plaque. Other variables, including the percentages of males and smokers, age, waist-hip ratio, SBP, DBP, and the creatinine level were also significantly different between groups. People with carotid plaque had a significantly thicker IMT than those without carotid plaque (0.80 mm vs 0.60 mm, *p* < 0.001).

**Table 2:** Characteristics of participants between groups with and without carotid plaque

Characteristics	Plaque (+) (*n* = 49)	Plaque (−) (*n* = 262)	^a^ *p*
Age (years)	61.90 ± 10.11	45.69 ± 11.67	< 0.001
Male, n (%)	25 (51.0)	86 (32.8)	0.015
BMI (kg/m^2^)	23.67(21.10, 26.57)	23.18 (20.90, 25.57)	0.408
WHR	0.93 ± 0.10	0.87 ± 0.08	< 0.001
Smoking, n (%)	16 (32.7%)	51 (19.5%)	0.039
Drinking, n (%)	13 (26.5%)	68 (26.0%)	0.933
FPG (mmol/L)	4.92 (4.57, 5.34)	4.81 (4.53, 5.15)	0.224
SBP (mmHg)	130.00 (115.67, 153.50)	111.33 (101.00, 125.25)	< 0.001
DBP (mmHg)	81.73 ± 10.74	74.90 ± 10.71	< 0.001
TC (mmol/L)	5.28 ± 1.14	4.68 ± 0.83	< 0.001
TG (mmol/L)	1.13 (0.82, 1.52)	1.01 (0.79, 1.55)	0.369
LDL-C (mmol/L)	3.07 (2.52, 3.86)	2.67 (2.23, 3.24)	0.002
HDL-C (mmol/L)	1.69 ± 0.40	1.58 ± 0.33	0.041
Non-HDL-C (mmol/L)	3.59 ± 1.14	3.10 ± 0.83	0.006
TC/HDL-C	3.17 (2.70, 3.86)	2.90 (2.53, 3.41)	0.065
LDL-C/HDL-C	1.97 (1.52, 2.43)	1.77 (1.40, 2.16)	0.059
ALT (IU/L)	18.00 (14.00, 23.00)	19.00 (15.00, 30.25)	0.173
AST (IU/L)	23.00 (19.50, 28.00)	22.00 (18.00, 27.00)	0.412
BUN (mmol/L)	5.60 (4.88, 6.82)	5.37 (4.30, 6.50)	0.118
Cr (umol/L)	60.72 ± 17.56	54.45 ± 13.75	0.006
IMT (mm)	0.80 (0.70, 0.90)	0.60 (0.60, 0.70)	< 0.001

^a^Student’s t-test, Mann-Whitney U test or Chi-square test

*BMI* Body mass index, *WHR* Waist-hip ratio, *FPG* Fasting plasma glucose, *SBP* Systolic blood pressure, *DBP* Diastolic blood pressure, *TC* Total cholesterol, *TG* Triglyceride, *LDL-C* Low-density lipoprotein cholesterol, *HDL-C* High-density lipoprotein cholesterol, *Non-HDL-C* Non-high-density lipoprotein cholesterol, *ALT* Alanine aminotransferase, *AST* Aspartate aminotransferase, *BUN* Blood urea nitrogen, *Cr* Creatinine, *IMT* Intima-media thickness

### Characteristics classified based on the dichotomized carotid IMT with a cutoff level of 0.9 mm

Based on the cutoff level of 0.9 mm, the percentage of people with a thickened IMT was 8.4%. As demonstrated in Table 3, age, waist-hip ratio, FPG, SBP, TC, LDL-C, non-HDL-C, TC/HDL-C ratio and LDL-C/HDL-C ratio were significantly different between the normal and thickened IMT groups.

**Table 3:** Characteristics of participants between groups with normal and thickened intima-media thickness

Characteristics	Normal (IMT ≤ 0.9 mm) (*n* = 285)	Thickened (IMT > 0.9 mm) (n = 26)	^a^ *p*
Age (years)	47.02 ± 12.45	61.69 ± 9.28	< 0.001
Male, n (%)	100(35.1%)	11 (42.3%)	0.602
BMI (kg/m^2^)	23.19(20.83, 25.64)	23.93 (22.46, 26.90)	0.073
WHR	0.87 ± 0.08	0.94 ± 0.08	< 0.001
Smoking, n (%)	63(22.1%)	4(15.4%)	0.583
Drinking, n (%)	74 (26.0%)	7 (26.9%)	0.915
FPG (mmol/L)	4.80 (4.53, 5.13)	5.19 (4.76, 5.61)	< 0.001
SBP (mmHg)	112.00 (101.33, 127.00)	135.33 (118.83, 144.50)	< 0.001
DBP (mmHg)	75.69 ± 11.02	79.09 ± 10.16	0.131
TC (mmol/L)	4.73 ± 0.88	5.26 ± 1.13	0.005
TG (mmol/L)	1.02(0.80, 1.51)	1.30 (0.82, 1.85)	0.196
LDL-C (mmol/L)	2.71 (2.26, 3.27)	3.15 (2.56, 3.71)	0.009
HDL-C (mmol/L)	1.60 ± 0.33	1.61 ± 0.41	0.870
Non-HDL-C (mmol/L)	3.13 ± 0.88	3.65 ± 1.02	0.005
TC/HDL-C	2.92(2.51, 3.44)	3.16 (2.86, 3.60)	0.034
LDL-C/HDL-C	1.77(1.38, 2.22)	1.89 (1.75, 2.39)	0.032
ALT (IU/L)	19.00 (15.00, 28.50)	17.50 (14.75, 24.00)	0.317
AST (IU/L)	22.00 (19.00, 27.50)	22.00 (19.00, 26.00)	0.902
BUN (mmol/L)	5.37 (4.31, 6.53)	5.56 (4.97, 6.94)	0.195
Cr (umol/L)	55.05 ± 14.74	59.74 ± 11.81	0.116

^a^Student’s t-test, Mann-Whitney U test or Chi-square test

*IMT* Intima-media thickness, *BMI* Body mass index, *WHR* Waist-hip ratio, *FPG* Fasting plasma glucose, *SBP* Systolic blood pressure, *DBP* Diastolic blood pressure, *TC* Total cholesterol, *TG* Triglyceride, *LDL-C* Low-density lipoprotein cholesterol, *HDL-C* High-density lipoprotein cholesterol, *Non-HDL-C* Non-high-density lipoprotein cholesterol, *ALT* alanine aminotransferase, *AST* Aspartate aminotransferase, *BUN* Blood urea nitrogen, *Cr* Creatinine

### Spearman rank correlations between lipid parameters, other variables and carotid IMT

**Fig. 1:**
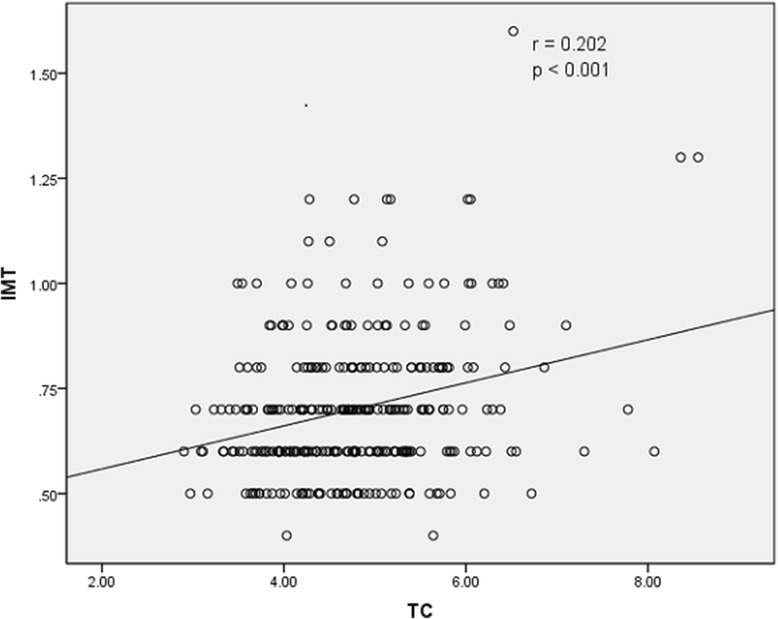
Correlation of intima-media thickness with TC level. TC = total cholesterol, IMT = intima-media thickness

**Fig. 2:**
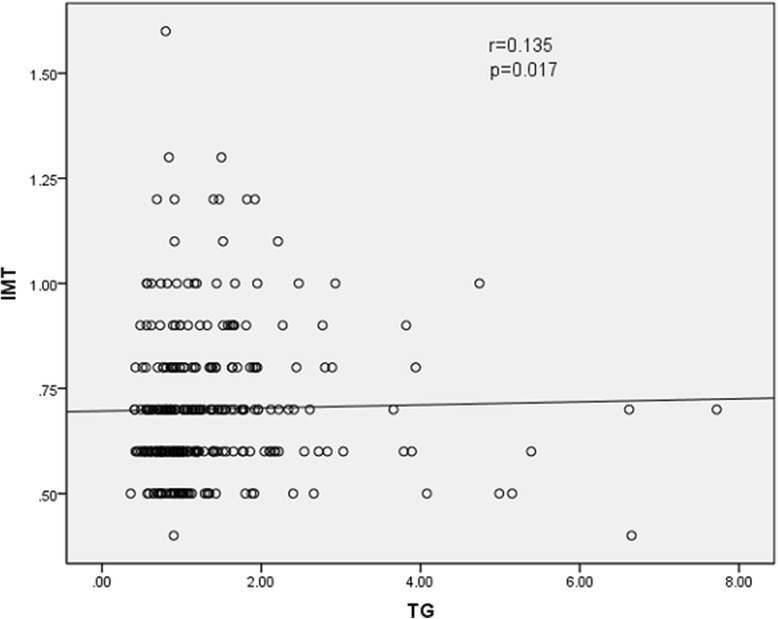
Correlation of intima-media thickness with TG level. TG = triglyceride, IMT = intima-media thickness

**Fig. 3:**
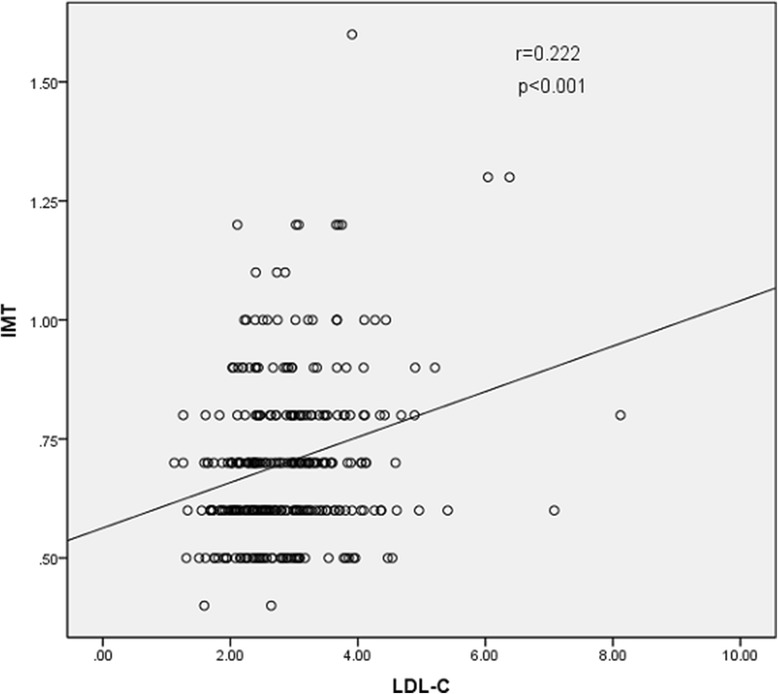
Correlation of intima-media thickness with LDL-C level. LDL-C = low-density lipoprotein cholesterol, IMT = intima-media thickness

**Fig. 4:**
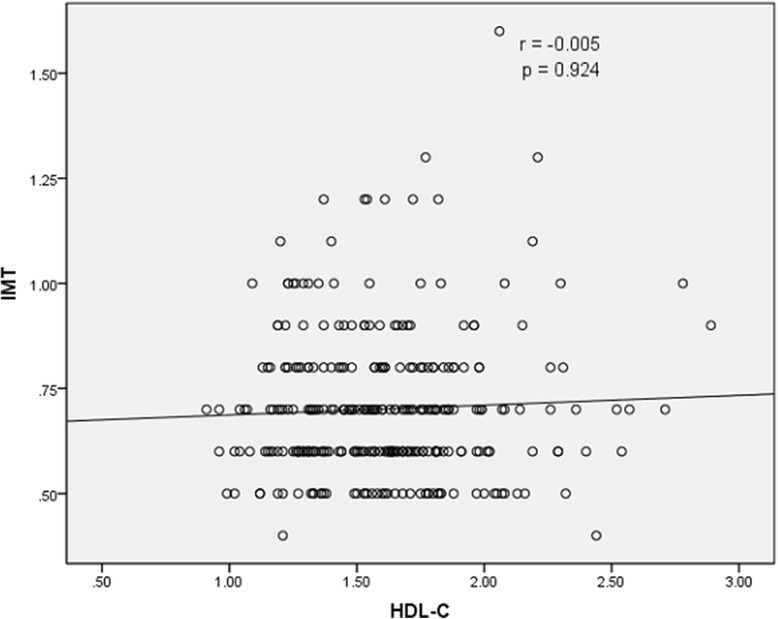
Correlation of intima-media thickness with HDL-C level. HDL-C = high-density lipoprotein cholesterol, IMT = intima-media thickness

**Fig. 5:**
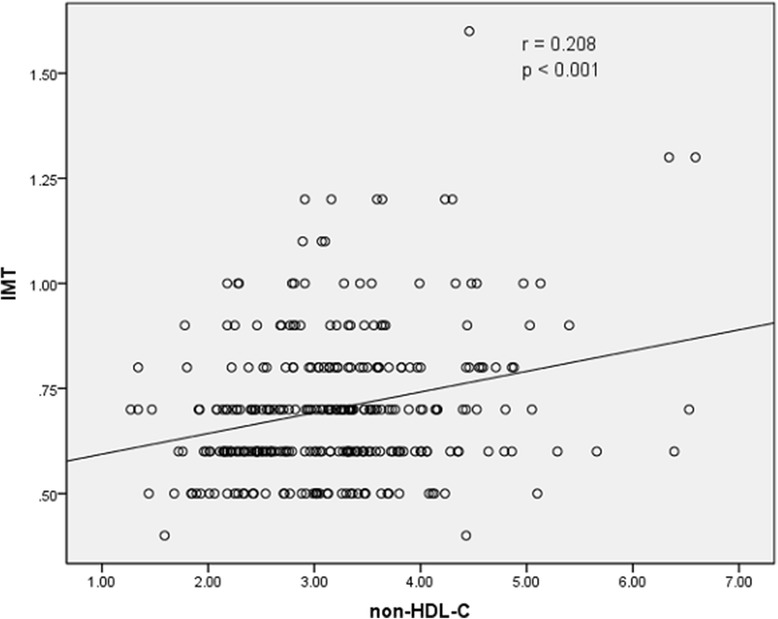
Correlation of intima-media thickness with non-HDL-C level. Non-HDL-C = non-high-density lipoprotein cholesterol, IMT = intima-media thickness

**Fig. 6:**
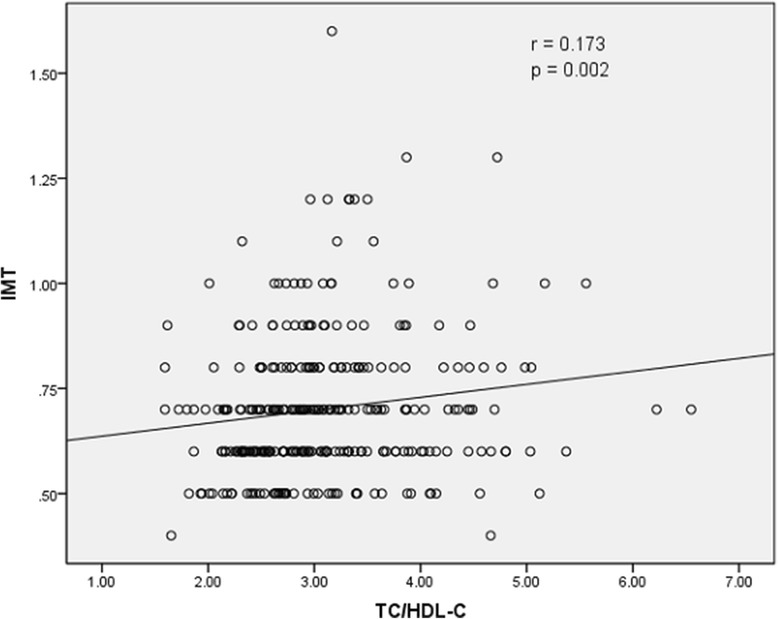
Correlation of intima-media thickness with TC/HDL-C ratio. TC = total cholesterol, HDL-C = high-density lipoprotein cholesterol, IMT = intima-media thickness

**Fig. 7:**
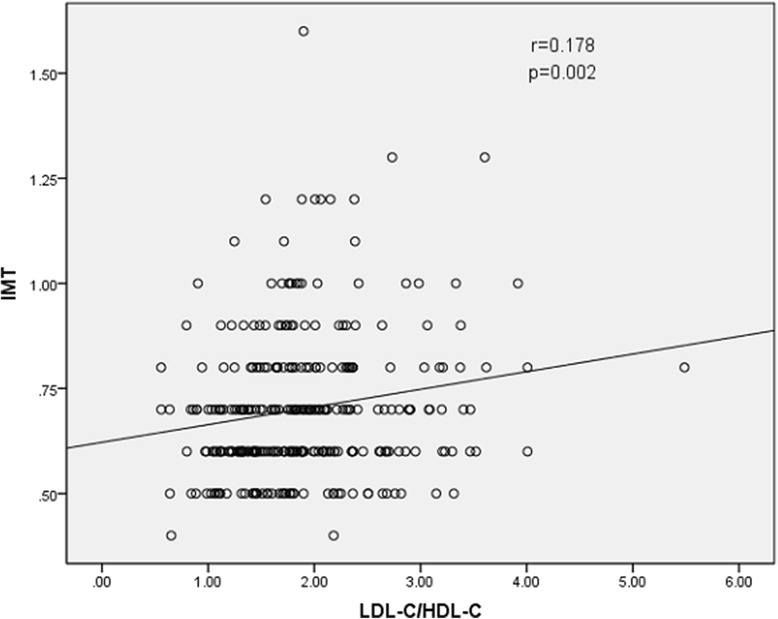
Correlation of intima-media thickness with LDL-C/HDL-C ratio. LDL-C = low-density lipoprotein cholesterol, HDL-C = high-density lipoprotein cholesterol, IMT = intima-media thickness

The results of Spearman rank correlation analyses are exhibited in Table 4. Among the lipid parameters, carotid IMT was positively correlated with the levels of LDL-C, TC and non-HDL-C (*r* = 0.222, 0.202 and 0.208, respectively; all *p* < 0.05) and the ratios of TC/HDL-C and LDL-C/HDL-C (*r* = 0.173 and 0.178, respectively; both *p* < 0.05) (Table 4 and Figs. 1, 2, 3, 4, 5, 6 and 7). Among other variables, carotid IMT was significantly correlated with age (*r* = 0.566, *p* < 0.001), gender (*r* = − 0.204, *p* < 0.001), BMI (*r* = 0.232, *p* < 0.001), waist-hip ratio (*r* = 0.372, *p* < 0.001), smoking (*r* = 0.130, *p* = 0.021), FPG (*r* = 0.217, *p* < 0.001), SBP (*r* = 0.427, *p* < 0.001), DBP (*r* = 0.272, *p* < 0.001), BUN (*r* = 0.150, *p* = 0.008) and creatinine (*r* = 0.238, *p* < 0.001) (Table 4).

**Table 4:** Spearman rank correlations between lipid parameters, other variables and carotid intima-media thickness

Variables	r	^a^ *p*
Age	0.566	< 0.001
Gender	−0.204	< 0.001
BMI	0.232	< 0.001
WHR	0.372	< 0.001
Smoking	0.130	0.021
Drinking	0.098	0.084
FPG	0.217	< 0.001
SBP	0.427	< 0.001
DBP	0.272	< 0.001
TC	0.202	< 0.001
TG	0.135	0.017
LDL-C	0.222	< 0.001
HDL-C	−0.005	0.924
Non-HDL-C	0.208	< 0.001
TC/HDL-C	0.173	0.002
LDL-C/HDL-C	0.178	0.002
ALT	−0.051	0.368
AST	0.052	0.364
BUN	0.150	0.008
Cr	0.238	< 0.001

^a^Spearman rank correlation test

*BMI* Body mass index, *WHR* Waist-hip ratio, *FPG* Fasting plasma glucose, *SBP* Systolic blood pressure, *DBP* Diastolic blood pressure, *TC* Total cholesterol, *TG* Triglyceride, *LDL-C* Low-density lipoprotein cholesterol, *HDL-C* High-density lipoprotein cholesterol, *Non-HDL-C* Non-high-density lipoprotein cholesterol, *ALT* Alanine aminotransferase, *AST* Aspartate aminotransferase, *BUN* Blood urea nitrogen, *Cr* Creatinine

### Univariate and multivariate linear regression analyses for correlations with carotid IMT

The results of univariate linear regression analyses are revealed in Table 5. Among the lipid parameters, the levels of TC (standard Beta = 0.252, *p* < 0.001), non-HDL-C (standard Beta = 0.243, *p* < 0.001) and LDL-C (standard Beta = 0.242, *p* < 0.001) appeared to be more closely related to carotid IMT compared with other lipid parameters in the univariate model. Subsequently, stepwise multiple linear regression analysis was performed using all the variables with a significance level of < 0.05 in the univariate linear regression analyses as independent variables and the log transformed carotid IMT as the dependent variable. The final results revealed that age (standard Beta = 0.457, *p* < 0.001), gender (standard Beta = − 0.101, *p* = 0.029) and SBP (standard Beta = 0.182, *p* = 0.001) were included in the multivariate model and remained as independent variables those were associated with carotid IMT (Table 6).

**Table 5:** Associations between lipid parameters, other variables and carotid intima-media thickness

Variables	Beta	Standard Beta	^a^ *p*
Age	0.004	0.568	< 0.001
Gender	−0.034	−0.167	0.003
BMI	0.006	0.230	< 0.001
WHR	0.444	0.385	< 0.001
Smoking	0.027	0.112	0.048
FPG	0.009	0.145	0.010
SBP	0.002	0.453	< 0.001
DBP	0.002	0.273	< 0.001
TC	0.027	0.252	< 0.001
TG	0.001	0.014	0.805
LDL-C	0.026	0.242	< 0.001
HDL-C	0.009	0.031	0.587
Non-HDL-C	0.026	0.243	< 0.001
TC/HDL-C	0.018	0.143	0.011
LDL-C/HDL-C	0.024	0.173	0.002
BUN	0.002	0.066	0.249
Cr	0.001	0.190	0.001

^a^Univariate linear regression analysis

*BMI* Body mass index, *WHR* Waist-hip ratio, *FPG* Fasting plasma glucose, *SBP* Systolic blood pressure, *DBP* Diastolic blood pressure, *TC* Total cholesterol, *TG* Triglyceride, *LDL-C* Low-density lipoprotein cholesterol, *HDL-C* High-density lipoprotein cholesterol, *Non-HDL-C* Non-high-density lipoprotein cholesterol, *ALT* Alanine aminotransferase, *AST* Aspartate aminotransferase, *BUN* Blood urea nitrogen, *Cr* Creatinine

**Table 6:** Predictors for carotid intima-media thickness by multiple linear regression analysis

Variables	Beta	Standardized Beta	^a^ *p*
Age	0.003	0.457	< 0.001
Gender	−0.021	−0.101	0.029
SBP	0.001	0.182	0.001

^a^Stepwise multiple linear regression analysis

*SBP* Systolic blood pressure

### Univariate and multivariate logistic regression analyses for correlations with the presence of carotid plaque

The results of univariate logistic regression analyses are shown in Table 7. Among the atherogenic lipids, the levels of TC (OR = 1.949, 95% CI = 1.403–2.709, *p* < 0.001), non-HDL-C (OR = 1.749, 95% CI = 1.265–2.418, *p* = 0.001) and LDL-C (OR = 1.616, 95% CI = 1.187–2.201, *p* = 0.002) appeared to be more closely related to carotid plaque in the univariate model. Stepwise multiple logistic regression analysis was conducted using all the variables with a significance level of < 0.05 in the univariate model as independent variables and the presence of carotid plaque as the dependent variable. As shown in Table 8, gender (OR = 2.216, 95% CI = 1.044–4.705, *p* = 0.038), TC level (OR = 1.568, 95% CI = 1.055–2.331, *p* = 0.026), age (OR = 1.127, 95% CI = 1.086–1.171, *p* < 0.001) and DBP (OR = 1.038, 95% CI = 1.002–1.075, *p* = 0.037) were independently associated with the presence of carotid plaque in the multivariate model.

**Table 7:** Associations between lipid parameters, other variables and the presence of carotid plaque

Variables	B	OR /EXP (B)	95% CI	^a^ *p*
Age	0.120	1.127	1.089–1.166	< 0.001
Gender	0.757	2.132	1.151–3.949	0.016
BMI	0.022	1.022	0.939–1.112	0.613
WHR	8.299	4018.118	80.940–199,473.238	< 0.001
Smoking	−0.696	0.499	0.255–0.975	0.042
Drinking	−0.030	0.971	0.486–1.939	0.933
FBG	0.074	1.076	0.917–1.264	0.368
SBP	0.039	1.039	1.025–1.055	< 0.001
DBP	0.054	1.056	1.027–1.085	< 0.001
TC	0.668	1.949	1.403–2.709	< 0.001
TG	−0.130	0.878	0.609–1.265	0.484
LDL-C	0.480	1.616	1.187–2.201	0.002
HDL-C	0.885	2.423	1.029–5.705	0.043
Non-HDL-C	0.559	1.749	1.265–2.418	0.001
TC/HDL-C	0.292	1.339	0.936–1.916	0.111
LDL-C/HDL-C	0.390	1.477	0.983–2.221	0.061
ALT	−0.024	0.977	0.952–1.002	0.073
AST	−0.003	0.997	0.960–1.305	0.864
BUN	0.002	1.002	0.929–1.081	0.961
Cr	0.030	1.030	1.008–1.052	0.006

^a^Univariate logistic regression analysis

*OR* Odds ratio, *CI* Confidence interval, *BMI* Body mass index, *WHR* Waist-hip ratio, *FPG* Fasting plasma glucose, *SBP* Systolic blood pressure, *DBP* Diastolic blood pressure, *TC* Total cholesterol, *TG* Triglyceride, *LDL-C* Low-density lipoprotein cholesterol, *HDL-C* High-density lipoprotein cholesterol, *Non-HDL-C* Non-high-density lipoprotein cholesterol, *ALT* Alanine aminotransferase, *AST* Aspartate aminotransferase, *BUN* Blood urea nitrogen, *Cr* Creatinine

**Table 8:** Predictors for carotid plaque by multivariate logistic regression analysis

Variables	OR	95% CI	^a^ *p*
Age	1.127	1.086–1.171	< 0.001
Gender	2.216	1.044–4.705	0.038
DBP	1.038	1.002–1.075	0.037
TC	1.568	1.055–2.331	0.026

^a^Multivariate logistic regression analysis

*OR* Odds ratio, *CI* Confidence interval, *DBP* Diastolic blood pressure, *TC* Total cholesterol

### The independent association of non-HDL-C level with carotid plaque by logistic regression analyses

Non-HDL-C level was associated with the presence of carotid plaque (OR = 1.749, 95% CI = 1.265–2.418, *p* = 0.001) (Table 7). And this association was tested by several models after adjusting for some conventional risk factors. As displayed in Table 9, high non-HDL-C level remained as a positive predictor for carotid plaque independent of age, gender, waist-hip ratio, smoking, drinking, SBP and FPG (OR = 1.565, 95% CI = 1.053–2.327, *p* = 0.027) (Table 9).

**Table 9:** Association of non-high-density lipoprotein cholesterol level with carotid plaque after adjusting for clinical variables

Adjustment	OR	95%CI	^a^ *p*
Non-HDL-C	1.749	1.265–2.418	0.001
Adjust for age, gender	1.526	1.047–2.223	0.028
Adjust for age, gender, WHR	1.555	1.061–2.278	0.024
Adjust for age, gender, WHR, smoking, drinking	1.543	1.045–2.277	0.029
Adjust for age, gender, WHR, smoking, drinking, SBP	1.526	1.031–2.260	0.035
Adjust for age, gender, WHR, smoking, drinking, SBP, FPG	1.565	1.053–2.327	0.027

^a^Logistic regression analysis

*OR* Odds ratio, *CI* Confidence interval, *non-HDL-C* Non-high-density lipoprotein cholesterol, *WHR* Waist-hip ratio, *SBP* Systolic blood pressure, *FPG* Fasting plasma glucose

## Discussion

The present study has demonstrated that age, gender and SBP are important predictors for carotid IMT. Age, gender, DBP and TC are important predictors for carotid plaque. TC, LDL-C and non-HDL-C have greater predictive values for carotid IMT and plaque compared with other lipid parameters, among which TC has the greatest predictive value for the presence of carotid plaque. The predictive value of non-HDL-C for carotid IMT and plaque is not inferior to that of LDL-C.

Atherogenesis is a complex process of several pathological stages: intimal medial thickening, fatty streaks, intermediate lesions, fibrous plaques, complicated plaques [13]. These different stages of atherogenesis depend on endothelial dysfunction, oxidative stress and inflammation. Many atherosclerotic risk factors can influence the levels of oxidative stress and inflammation in vivo or directly impair the endothelial function of the vessel wall.

Advancing age is an important predictor for carotid IMT and the presence of carotid plaque. Bauer et al. have reported that almost 50 to 80% of increased carotid IMT is attributed to aging and the carotid IMT of men increases by 0.007 mm per year [14]. The underlying pathogenesis can be explained by age-related impairment of endothelial function. The endothelium produces endothelium-derived relaxing factors such as nitric oxide that protects the vessel wall from developing atherosclerosis. With increasing age, the endothelium-derived relaxing factors decrease and the endothelium-derived vasoconstrictor factors increase. In addition, the generation of reactive oxygen species (ROS) increases with advanced age. ROS cause nitric oxide break down and consequently impair endothelial function of the vessel wall [15].

Male gender is another important predictor for carotid IMT and the presence of carotid plaque. Previous studies have demonstrated that there is a gender difference in the cardiovascular morbidity and mortality. Premenopausal females are at lower risk of CVD than males and this gender difference disappears between postmenopausal females and males. Moreover, premature menopausal females are at increased risk of CVD compared with premenopausal females of the same age. These results suggest that estrogens play a vital role in the gender difference of atherosclerosis risk. Estrogens can stimulate the release of endothelium-derived relaxing factor and inhibit the rennin-angiotensin system (RAS) [16, 17], therefore protecting the endothelial function of the vessel wall.

High blood pressure can induce loss of vasomotor activity and perpetuate endothelial damage, thus triggering a wide range of different vasculopathies including increasing lipid permeability, promoting oxidative stress and activating renin-angiotensin system (RAS) and sympathetic nervous system (SNS) [18]. Several previous studies have revealed the correlation between blood pressure and carotid IMT or plaque [19–21]. In the present study, multivariate analyses have demonstrated that SBP is correlated with carotid IMT, while DBP is correlated with the presence of carotid plaque. It might mean SBP is a better predictor of early atherosclerosis and DBP is a better predictor of advanced atherosclerosis. However, several other explanations for this difference should also be taken into consideration: first, the sample size of this study is relatively limited; second, the characteristics of the subjects in the present study are not the same as the ones in the previous studies; third, the resolution of the current ultrasound images might be inadequate to distinguish between medial hypertrophy (pathogenesis of hypertension) and intimal thickening (pathogenesis of atherosclerosis). So further study with a large sample size based on high-resolution measurements is needed.

Compared with other lipid parameters, TC has the greatest predictive value for the presence of carotid plaque. Previous prospective studies have proved that there is a continuous, graded and strong relationship between serum TC level and six-year age-adjusted risk of CHD death [22]. Compared with young men aged 18 to 39 years with favorable TC levels (TC < 5.17 mmol/L), those with unfavorable TC levels (TC ≥ 6.21 mmol/L) had a greater risk of CHD (2.15 to 3.63 times) and CVD mortality (2.10 to 2.87 times) [23]. In this study, LDL-C, which is a classical atherogenic lipid, has a lower predictive value for the presence of carotid plaque than TC. This discrepancy may partly be explained by the following reasons. First, in addition to LDL-C, very low-density lipoprotein cholesterol (VLDL-C) and intermediate-density lipoprotein cholesterol (IDL-C) are also closely related to atherosclerosis. Second, the baseline mean TG level of the participants was 1.03 mmol/L, so most LDL-C levels were calculated by the Friedwald eq. (LDL-C = TC-HDL-C-TG/2.2) in this study. Hendrick et al. have found that when TG < 2.26 mmol/L, the overall misclassification rate of CVD risk score ranged from 5 to 17% by using calculated LDL-C [24]. Third, the baseline mean LDL-C level in this study was nearly normal (2.78 mmol/L). Previous studies have revealed that high LDL-C level is a risk factor for atherosclerosis, while low or normal LDL-C level is well tolerated by individuals [25].

The predictive value of non-HDL-C for carotid IMT and plaque is not inferior to that of LDL-C. Multivariate analysis demonstrated that non-HDL-C remained as a positive predictor for carotid plaque after adjustment of age, gender, waist-hip ratio, smoking, drinking, SBP and FPG. Our results are consistent with the previous findings. Previous published epidemic surveys have shown that non-HDL-C is a somewhat better predictor for CVD risk than LDL-C [5, 26]. Framingham Heart Study (FHS) has found that non-HDL-C appears to be a stronger predictor of CVD risk than LDL-C independent of TG levels [27, 28]. Another meta-analysis of more than 300,000 people has demonstrated that the predictive value of non-HDL-C for CVD risk is not inferior to that of LDL-C (both measured and calculated) [29]. Non-HDL-C as a risk predictor of atherosclerosis has several advantages: first, it contains all potential atherogenic lipid particles; second, it can be calculated in the non-fasting state; third, it can be calculated in the setting of hypertriglyceridemia.

The present study has revealed the predictors for carotid IMT and plaque in participants without the diagnoses of acute myocardial infarction, stroke, heart failure, or peripheral arterial disease. And as we all know, thickened carotid IMT and the presence of carotid plaque are surrogate markers for heart attack and stroke. Therefore, the results of this study will be meaningful for detecting early-stage asymptomatic atherosclerosis and preventing cardiovascular events in the general Chinese adults. The results of this study are relatively reliable because participants on lipid-lowering therapy were excluded. Moreover, we compared the predictive values of different lipid parameters for carotid IMT and plaque.

This study may have several potential limitations. Firstly, this is an observational study with a relatively small sample size. Only correlations rather than causality can be drawn due to the nature of the study design. Secondly, this is a single-center study and almost all the participants are Han Chinese from the same community, so we may not generalize the results to other regions or other ethnicities. Further multi-centered, prospective cohort studies with a larger sample size are needed. Moreover, the present study only detected the predictors for plaque presence due to the insufficient data. The predictors for the quantitative characteristics of carotid plaque such as plaque number, plaque thickness, plaque area, plaque type and the Doppler ultrasound blood flow signal deserve further investigation.

## Conclusions

In conclusion, the present study has revealed that age, gender and SBP are important predictors for carotid IMT. Age, gender, DBP and TC are important predictors for carotid plaque. TC, LDL-C and non-HDL-C have greater predictive values for IMT and the presence of carotid plaque compared with other lipid parameters. The predictive value of non-HDL-C for carotid IMT and plaque is not inferior to that of LDL-C.

## Abbreviations

ALT: Alanine aminotransferase
AST: Aspartate aminotransferase
BMI: Body mass index
BUN: Blood urea nitrogen
CHD: Coronary heart disease
CI: Confidence interval
Cr: Creatinine
CVD: Cardiovascular disease
DBP: Diastolic blood pressure
DM: Diabetes mellitus
FHS: Framingham heart study
FPG: Fasting plasma glucose
HDL-C: High-density lipoprotein cholesterol
IDL-C: Itermediate-density lipoprotein cholesterol
IMT: Intima-media thickness
LDL-C: Low-density lipoprotein cholesterol
Non-HDL-C: Non-high-density lipoprotein cholesterol
OR: Odds ratio
RAS: Rennin-angiotensin system
ROS: Ractive oxygen species
SBP: Systolic blood pressure
SD: Standard deviation
SNS: Sympathetic nervous system
TC: Total cholesterol
TG: Triglyceride
VLDL-C: Very low-density lipoprotein cholesterol
WHR: Waist-hip ratio
